# A critical evaluation of the status of HPV vaccination in São Paulo State, Brazil

**DOI:** 10.1016/j.clinsp.2024.100526

**Published:** 2024-11-06

**Authors:** Toni Ricardo Martins, Steven S. Witkin, Andressa da Silva Ferreira, Juliana Yukari K. Viscondi, Maryana Stephany Ferreira Branquinho, Lise Cury, Lucy Santos Vilas Boas, Adhemar Longatto-Filho, Maria Cássia Mendes-Corrêa

**Affiliations:** a Faculdade de Ciências Farmacêuticas da Universidade Federal do Amazonas, Manaus, AM, Brazil; b Programa de Pós graduação em Imunologia Básica e aplicada – PPGIBA – Universidade Federal do Amazonas, Manaus, Brasil; c Department of Obstetrics and Gynecology, Weill Cornel Medicine, New York, USA; d Medicine Tropical Institute, Virology Laboratory ‒ LIM/52, Faculdade de Medicina da Universidade de São Paulo, São Paulo, SP, Brazil; e Undergraduate Biomedicine Course at Universidade Paulista, São Paulo, SP, Brazil; f Health Technology Assessment Unit, Oswaldo Cruz German Hospital (HAOC), São Paulo, SP, Brazil; g Biomedicine Course at Universidade Paulista, São Paulo, SP, Brazil; h Fundação Oncocentro de São Paulo (FOSP), São Paulo, SP, Brazil; i Life and Health Sciences Research Institute (ICVS), School of Medicine, University of Minho, Braga, Portugal; j ICVS/3B's – PT Government Associate Laboratory, Braga/Guimarães, Portugal; k Medical Laboratory of Medical Investigation (LIM) 14, Department of Pathology, Faculdade de Medicina da Universidade de São Paulo, São Paulo, SP, Brazil; l Molecular Oncology Research Center, Hospital de Câncer de Barretos, São Paulo, SP, Brazil; m Infectious Diseases Department, Faculdade de Medicina da Universidade de São Paulo, São Paulo, SP, Brazil

**Keywords:** HPV vaccine, HPV, Papanicolaou Test, Cervical Cancer

## Abstract

Cervical cancer, whose well-recognized etiological agent is the Human Papillomavirus (HPV), is responsible for approximately 300,000 deaths worldwide, 80% of cases occurring in developing countries. In Brazil, 17,010 cases were expected, with 2,550 cases in São Paulo State, in 2023. The Papanicolaou test is the diagnostic method for the detection of precancerous lesions of the cervix, and HPV vaccination is now available for prevention.

**Materials and methods:**

This is a descriptive and exploratory, retrospective investigation, carried out through analysis of data obtained from Brazilian Information Technology (DATASUS) of the Brazilian Federal Government: Cancer Information System (SISCAN), Brazilian National Immunization Program (PNI) and Mortality Information System (SIM). Electronic Library and data made available by the Government of the State of São Paulo.

**Results:**

The number of women in São Paulo State who underwent cytological examinations and histological tests for cervical cancer decreased between the Years 2013 and 2022.

**Conclusion:**

The continuous increase in cervical cancer over the study period was probably due to the lack of adherence to the primary and secondary prevention opportunities offered by the Public Health Authorities.

## Highlights

• In Brazil, 17,010 cases of cervical cancer were expected, with 2550 cases in São Paulo State in 2023.
• The number of women in São Paulo State who underwent cytological examinations and histological tests for cervical dysbiosis, or cancer deceased between the years 2013 and 2022.
• The number of HPV vaccinations and the doses administered also declined.
• The continuous increase in cervical cancer over the study period was probably due to the lack of adherence to the primary and secondary prevention opportunities offered by the Public Health Authorities.
• Improved efforts by the Public Health System are needed in association with the implementation of current medical guidelines to reduce this shortcoming.

## Introduction

In 2022, cervical cancer was responsible for approximately 340,000 deaths worldwide, more than 90% occurring in developing countries (https://www.iarc.who.int/cancer-type/cervical-cancer/, November 14, 2023). In Brazil, it was estimated that 17,010 cases will be diagnosed in 2023, with 2550 cases occurring in the State of São Paulo (https://www.gov.br/inca/pt-br).

Human Papillomavirus (HPV) infection is mainly transmitted through unprotected sexual contact and can remain silent without any signs or symptoms. It is a DNA virus that infects the squamous epithelium (skin and mucous membranes), causing the appearance of anogenital warts and/or malignant transformation in the cervical region, depending on the genotype of the infecting virus.^1^^,^^2^ HPV represents a group of very ancient viruses that have lived with humans for millennia.^3^ It has been documented that HPV can replicate and survive in anatomical zones of the stratified epithelia. McBride's elegant review referenced that almost 450 HPV types have been currently sequenced, highlighting their enormous potential to replicate and induce benign and malignant neoplasias.^4^

The ability of HPV to escape the host's immune responses and to persist has created many questions regarding how to effectively prevent, diagnose, and treat infections by oncogenic HPV types.^5^ The Pap smear test has been advocated by Brazilian Public Health authorities as the first option for preventing cervical cancer by the early detection and subsequent treatment of precursor lesions in the general female population. However, the cytological performance of this test has been critically analyzed and further improvements are clearly necessary for it to reach acceptable quality parameters.^6^^,^^7^

The latest primary means of prevention of cervical cancer is by vaccination against oncogenic HPV types. The results of more than 20 years of HPV vaccination in many countries have validated its huge potential to prevent HPV-associated cervical malignancy, although its effectiveness appears to vary slightly according to the type of HPV employed in the vaccine.^8^ The Brazilian Unified Health System (SUS) offers the Quadrivalent vaccine, which protects against HPV genotypes 6, 11, 16 and 18. The vaccine is extremely effective in primary prevention and, therefore, it is essential to provide its dissemination to the general public. Education about the unequivocal benefits of vaccination, and combatting myths and fake news about adverse side effects is also essential.^9^ Brazilian data currently demonstrates low adherence to HPV vaccination in this country, probably due to the lack of continued educational outreach and appropriate social marketing, in contrast to its effective utilization in other countries.^10^

It has been established that vaccination carried out in a school environment, with the consent and participation of parents and educators, was an excellent option to increase adherence and also to educate about the importance of health of HPV vaccination.^11^ This practice, however, to the best of our knowledge, is not currently being encouraged in Brazil. The objective of the present study is to present a compilation of official data on HPV vaccination in the State of São Paulo and cervical cancer-related- mortality rates before, during, and after the introduction of the HPV vaccine.

## Materials and methods

### *Data acquisition*

This is a descriptive and exploratory, retrospective investigation, carried out through analysis of data obtained from access to multiple sources: the Brazilian Information Technology (DATASUS) of the Brazilian Federal Government, demographic information obtained in the database of the Brazilian Institute of Statistical Geography (IBGE), PUBMED (Service of the U.S. National Library of Medicine), LILACS (Latin American and Caribbean Literature in Health Sciences), SCIELO (Scientific Electronic Library) and data made available by the Government of the State of São Paulo.

### *Data analysis*

The data was organized and analyzed using Microsoft Excel software. Frequency, mean, and Standard Deviation (SD) calculations were performed.

## Results

**Fig. 1:**
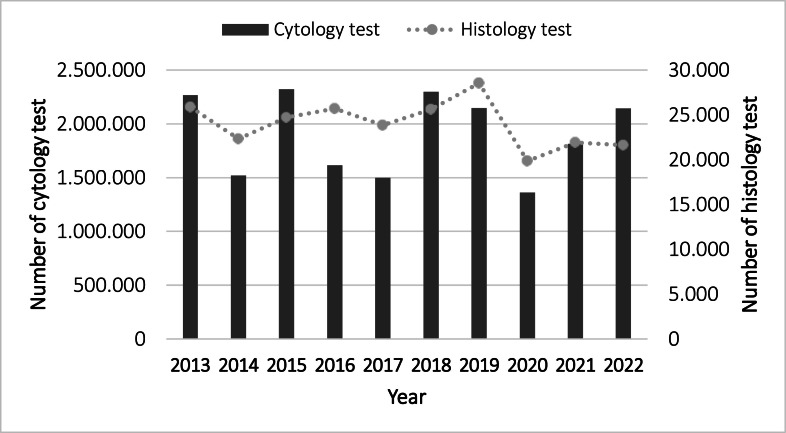
Number of women undergoing PAP smear cytological and histological examinations in São Paulo State, Brazil, from 2013 to 2022 (Source: SISCAN https://www.inca.gov.br/sites/ufu.sti.inca.local/files/media/document/siscan_modulo_1_2021.pdf).

Between 2013 to 2022, in the State of São Paulo a mean (SD) of 1898,383 (336,894) women underwent a yearly PAP smear cytological examination, in 2013, 2267,291 examinations were recorded. In 2014, when HPV immunization became available, there was a 33.1% decrease in PAP smears. This number increased 18% in 2015, followed by decreases of 25.1% in 2016 and 26.8% in 2017. In 2018 there was a 17.4% increase above the 2013 level. In 2019 the number of exams decreased slightly to 92.6% the 2013 level. With the arrival of the COVID-19 pandemic in 2020, there was the most pronounced decrease in PAP examinations (39.5%). In 2021 and 2022 the number of PAP procedures progressively increased to 79.5% and 90.4% of the 2013 level. The data for each year are summarized in Fig. 1.

Fig. 1 also displays the number of histological examinations performed in São Paulo State from 2013 to 2022. The mean (SD) annual number over this period was 24,012 (2094) examinations. The numbers were 25,882 in 2013, peaking at 15.9% above the average in 2019, dropping to 20.8% below the average in 2020 during the pandemic, then rising to 21,921 in 2021 and 21,623 in 2022.

**Fig. 2:**
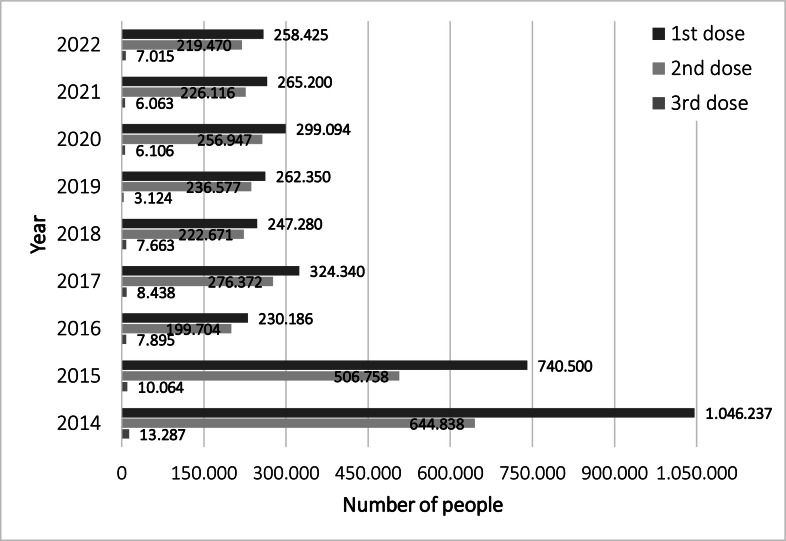
Number of HPV vaccine doses given in São Paulo State, Brazil, from 2014 to 2022 (Source: DATASUS: PNI).

Fig. 2 presents the number of women who received the HPV vaccine annually and by dose. In its debut year in 2014, 1046,237 women were vaccinated with the first dose. In the following year, there was a 29.2% decrease, followed by annual variations that maintained a decreasing trend, resulting in a mean (SD) of 269,554 (24.01) (74.2% less compared to the first year). Adherence to the second dose in the first two years was around 65.5%, while in the following years it reached approximately 87%. The adherence to the third dose was around 2.7%.

**Fig. 3:**
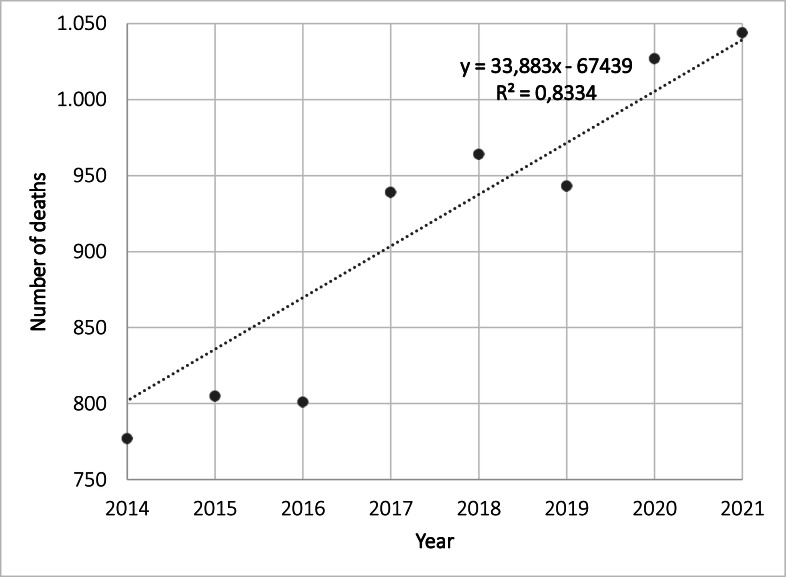
Number of deaths caused by malignant neoplasms in the cervix, in São Paulo State, Brazil, from 2013 to 2021 (Source: DATASUS: SIM).

Mortality data for women who had cervical cancer, in São Paulo State, was obtained from DATASUS, specifically focusing on the year 2013, prior to the availability of the HPV vaccine in Brazil, and subsequent years following its implementation for girls aged 11 to 13 (Fig. 3). Between the years 2013 and 2021, the mean (SD) number of yearly recorded deaths was 903 (89). There were 833 deaths recorded in 2013. Shortly after the vaccine became available, there was a 6.7% decrease the following year, but in subsequent years, there was a nearly linear increase, surpassing a thousand deaths after 2020 (the year of the pandemic).

## Discussion

Prevention of HPV-related lesions has been a priority in Brazil since 1998, when the *Viva Mulher* program was initiated.^12^ In the present study, the authors document that the number of women who sought medical care for HPV-related cytological and histological examinations before the availability of the HPV vaccine was very low. In 2014, with the arrival of the vaccine, along with the implementation of educational and promotional programs and recommendations, the number of women who underwent HPV evaluations increased. From 2016 to 2018 the number of examinations continued to grow. However, coinciding with the COVID pandemic in 2020 this number decreased^13^ and, in 2023, this decline continued. A recent report noted that the majority of women who present with high-grade HPV lesions in cytological and histological examinations are between 30 to 39 years of age or older.^14^ This is relevant because these women have had no opportunity to be vaccinated, and the consequences of this are apparent. In many women for whom the Pap test is not available, or who do not follow standard medical practices, directly testing for cervical HPV by molecular protocols is an alternative that can be repeated at 5-year intervals.^15^

The HPV vaccine has made significant progress in preventing high-grade HPV-induced lesions and also has substantially reduced the rate of HPV infection.^16^ The vaccine is highly effective against HPV-16 and −18, being able to prevent persistent infections and reduce the development of pre-cancerous lesions. Additionally, HPV vaccine efficacy was later demonstrated at non-cervical anogenital anatomic sites, including the vulva, vagina, pênis, and anus.^17^, ^18^, ^19^ The World Health Organization recommends vaccination for girls aged 9 to 14 years, as well as for individuals with an elevated risk of HPV-induced cancer.^20^ According to the US Centers for Disease Control and Prevention Advisory Committee on Immunization Practices routine HPV vaccination is recommended for females and males aged 9 to 26 and permissive vaccination based on shared clinical-decision making for individuals aged 27 to 45. The vast majority of countries are implementing female-only vaccination programs; currently, only 18% of the global HPV vaccine supply is being utilized by males. In 2020, the US FDA expanded its recommendation of the HPV vaccine ‒ HPV 9v (HPV types 6, 11, 16, 18, 31, 33, 45, 52 and 58) ‒ to include prevention of oropharyngeal and other head and neck cancers, including epithelial malignancies involving the upper respiratory/digestive tract: lips, oral cavity, oropharynx, nasal cavity, nasopharynx, hypopharynx and larynx/upper trachea). Similar approvals in other countries could further increase the utilization of gender-neutral HPV vaccination.^21^

In Brazil, there is a single health system (SUS), which provides the HPV vaccine free of charge, including the Quadrivalent vaccine, for girls aged 9 to 14, transplant recipients, cancer patients and individuals with HIV-related conditions. For girls aged 9 to 14, 2 doses are administered, with an interval of six months between them; in adolescents and adults from 15 years of age onwards, 3 doses are administered, the second at an interval of two months and the third dose applied 6 months after the second vaccination. Currently, The International Federation of Gynecology and Obstetrics recommends HPV vaccination for children as early as 9 years of age, and usually between 11 to 12 years, integrated into standard vaccination protocols for adolescents. Vaccinations prior to age 15 in adolescents are seen to be an essential pillar in reducing the incidence of cervical lesions and cancer.^22^ It is important to emphasize that, currently, one or two doses of the HPV vaccine are believed to be potentially effective for adolescents who have received the vaccine when under the age of 18.^23^

Recently, the Ministry of Health of Brazil initiated a new vaccination strategy against HPV. The scheme will now consist of a single dose, replacing the old model with two applications. The single dose recommendation was based on studies demonstrating with robust evidence the effectiveness of this regimen compared to two or three-stage vaccination regimes. Furthermore, the scheme follows the most recent recommendations from the World Health Organization and the Pan-American Health Organization.^24^ In addition to favorable scientific evidence, the single-dose strategy aims to increase vaccination adherence and expand vaccination coverage, aiming to eliminate cervical cancer as a public health problem. This is a measure in line with the World Health Organization's goal for the eradication of cervical cancer in the coming years.^25^

The HPV vaccine is recognized as a critical tool for preventing infection and related diseases, but adherence to vaccination campaigns in Brazil remains low in comparison to other countries. Vaccine coverage in Brazil is approximately 49.6% (2019 data), distant from countries like Mexico (97.5% in 2019), and Peru (91% in 2019).^10^ It is likely that most of the unimmunized Brazilian women do not understand the importance of immunization, or they do not have adequate information on the critical role of the HPV vaccine to avoid lesions that can progress to dysplastic alterations and cancer. Also, considering the utilization of private medical services for vaccination, it is plausible that many women do not have the financial means to undergo the recommended complete vaccination protocol. Additionally, the combination of lack of educational support by the Government lack of accessible recommendations, fake news dissemination, and lack of newspapers and television informative programs about the importance of the vaccine and all related benefits to preventing HPV types of infection and cervical cancer have probably been contributing to the under-utilization of the HPV vaccine.^26^

The results obtained to date indicate that the mortality rate for cervical cancer in the state of São Paulo continues to increase. It is worth mentioning, however, that a limitation in our study is the use of mortality data to assess the possible impact of vaccination. This may not be entirely appropriate as the program is still relatively new. The ideal would be to evaluate variation in the rates of early changes (CIN1, CIN2, or CIN3) in the age groups that have been covered by vaccination. In contrast, Australia which was one of the first countries to introduce a national HPV vaccination program, has achieved high vaccination coverage across both sexes. It has been estimated that cervical cancer could be considered to be eliminated as a public health problem in Australia within the next 20 years if high-coverage vaccination and screening are maintained at a threshold of four new cases per 100 000 women annually. By these criteria, cervical cancer could be considered to be eliminated as a public health problem in Australia within the next 20 years.^27^ Another consideration is that it is not totally clear of the correlation between the number of cytological examinations performed and vaccination coverage or even the vaccination program, since cervical cancer screening is recommended from 25 years of age and accession to the HPV vaccine begins at age 9. This makes it difficult in a country without any monitoring to trace this correlation. However, screening and vaccination initiatives would need to be ongoing thereafter to maintain a very low cervical cancer incidence and mortality rate. This is extremely important since cytological based screening must be carried out frequently with high adherence. So far, the results of Brazilian screening routines are still insufficient for women's safety and cervical cancer programs’ effectiveness.^6^^,^^7^ The dissemination of knowledge about HPV infection can play a critical role in promoting a more informed and compliant society. This will reduce disparities, encourage collaboration in programs and increase efficacy of campaigns to enhance public understanding of HPV's relationship with cancer. It will also provide support to women to take appropriate care and stimulate participation and continued screening adherence^28^ and encourage participation for those hesitant to permit their children's vaccination.^29^

In conclusion, the HPV vaccination program of the National Brazilian Program of Vaccination appears to be lacking in optimal efficacy. Similarly, São Paulo State is also deficient in the implementation of a program to vaccinate young girls and boys. Improved efforts by the Public Health System are needed in association with the implementation of current medical guidelines to reduce this shortcoming.

## Statement of ethics

The use of data, documents or records that are all publicly available (such as publicly accessible archives or publications) does not require ethical approval.

## Authors’ contributions

Conceptualization: Toni Ricardo Martins, Steven S. Witkin, Maria Cássia Mendes-Corrêa, and Adhemar Longatto-Filho.

Methodology: Toni Ricardo Martins, Steven S. Witkin, Andressa da Silva Ferreira, Juliana Visconde, Maryana Branquinho, Lise Cury, Lucy Vilas Boas, Adhemar Longatto-Filho and Maria Cássia Mendes-Corrêa.

Validation: Toni Ricardo Martins, Steven S. Witkin, Andressa da Silva Ferreira, Juliana Visconde, Maryana Branquinho, Lise Cury, Lucy Vilas Boas, Adhemar Longatto-Filho and Maria Cássia Mendes-Corrêa.

Writing-original draft: Toni Ricardo Martins, Steven S. Witkin, Andressa da Silva Ferreira, Adhemar Longatto-Filho and Maria Cássia Mendes-Corrêa.

Review and editing: Toni Ricardo Martins, Steven S. Witkin, Juliana Visconde, Adhemar Longatto-Filho and Maria Cássia Mendes Corrêa.

## Funding

The authors did not receive any funding during the study.

## Declaration of competing interest

The authors declare no conflicts of interest.
